# Using proteomics and metabolomics to identify therapeutic targets for senescence mediated cancer: genetic complementarity method

**DOI:** 10.3389/fendo.2023.1255889

**Published:** 2023-09-08

**Authors:** Xiaolu Fang, Deyang Liu, Jianzhong Zhao, Xiaojia Li, Ting He, Baishan Liu

**Affiliations:** ^1^ Department of Clinical Laboratory, Xiangyang No.1 People’s Hospital, Hubei University of Medicine, Xiangyang, China; ^2^ Department of Rehabilitation Medicine, Xiangyang No.1 People’s Hospital, Hubei University of Medicine, Xiangyang, China; ^3^ Department of Respiratory, Jiulongpo District People’s Hospital of Chongqing, Chongqing, China

**Keywords:** senescence, lung cancer, Network Mendelian randomization, proteomics and metabolomics, therapeutic target, genetic complementarity method senescence, genetic complementarity method

## Abstract

**Background:**

Senescence have emerged as potential factors of lung cancer risk based on findings from many studies. However, the underlying pathogenesis of lung cancer caused by senescence is not clear. In this study, we try to explain the potential pathogenesis between senescence and lung cancer through proteomics and metabonomics. And try to find new potential therapeutic targets in lung cancer patients through network mendelian randomization (MR).

**Methods:**

The genome-wide association data of this study was mainly obtained from a meta-analysis and the Transdisciplinary Research in Cancer of the Lung Consortium (TRICL), respectively.And in this study, we mainly used genetic complementarity methods to explore the susceptibility of aging to lung cancer. Additionally, a mediation analysis was performed to explore the potential mediating role of proteomics and metabonomics, using a network MR design.

**Results:**

GNOVA analysis revealed a shared genetic structure between HannumAge and lung cancer with a significant genetic correlation estimated at 0.141 and 0.135, respectively. MR analysis showed a relationship between HannumAge and lung cancer, regardless of smoking status. Furthermore, genetically predicted HannumAge was consistently associated with the proteins C-type lectin domain family 4 member D (CLEC4D) and Retinoic acid receptor responder protein 1 (RARR-1), indicating their potential role as mediators in the causal pathway.

**Conclusion:**

HannumAge acceleration may increase the risk of lung cancer, some of which may be mediated by CLEC4D and RARR-1, suggestion that CLEC4D and RARR-1 may serve as potential drug targets for the treatment of lung cancer.

## 1 Introduction

In recent research, a new multi-system based aging measurement method, called epigenic clock, has been widely recognized as a potential biological marker of senescence (1). Horvath et al. reported that the epigenetic clock is a measure of organismal aging that is indicated by DNA methylation (DNAm) patterns, which exhibit heritability. Each clock has unique DNAm levels at corresponding CpG sites, which reveal key features of age-related genetic changes (2). The first generation of epigenetic clocks, such as HannumAge and endogenous HorvathAge, were developed based on the DNAm levels of age-related CpG sites (3, 4). HannumAge uses a model trained on 71 age-related CpG sites found in blood samples, while endogenous HorvathAge selects from 353 CpG sites discovered in human tissues and cells, and corrects for differences in blood cell counts (3). Recently, second-generation clocks such as PhenoAge and GrimAge have been used to predict the epidemiological information related to age-related diseases (1, 5). PhenoAge was developed based on data from 513 CpG sites associated with mortality and 9 clinical biomarkers, while GrimAge incorporates data from 1030 CpG sites associated with smoking and 7 blood plasma proteins including cystatin C and growth differentiation factor (6). Depending on the sample source and prediction requirements, HannumAge is better suited for age-related predictions while PhenoAge and GrimAge are more suitable for health-related predictions (3, 7).

The relationship between epigenetic clocks and diseases is not yet fully understood. However, observational studies have found that when epigenetic age accelerates, meaning that a person’s biological age is greater than their chronological age, it may lead to increased mortality and cancer incidence (8, 9). For example, Levine et al. analyzed a dataset from the Women’s Health Initiative that included 2029 women and found that standardized measurements of intrinsic epigenetic age acceleration were significantly associated with increased cancer incidence (HR: 1.50, *P* = 3.4×10^−3^) (10). In contrast, some argue that the evidence supporting this claim is weak or nonexistent. For instance, Dugué et al. used Cox regression analysis to examine the relationship between five measures of age acceleration and 3216 cancer patients, finding no association between the two (11). The primary reason for the controversy may be due to the biases of observational studies, such as reverse causality and residual confounding.

**Figure 1:**
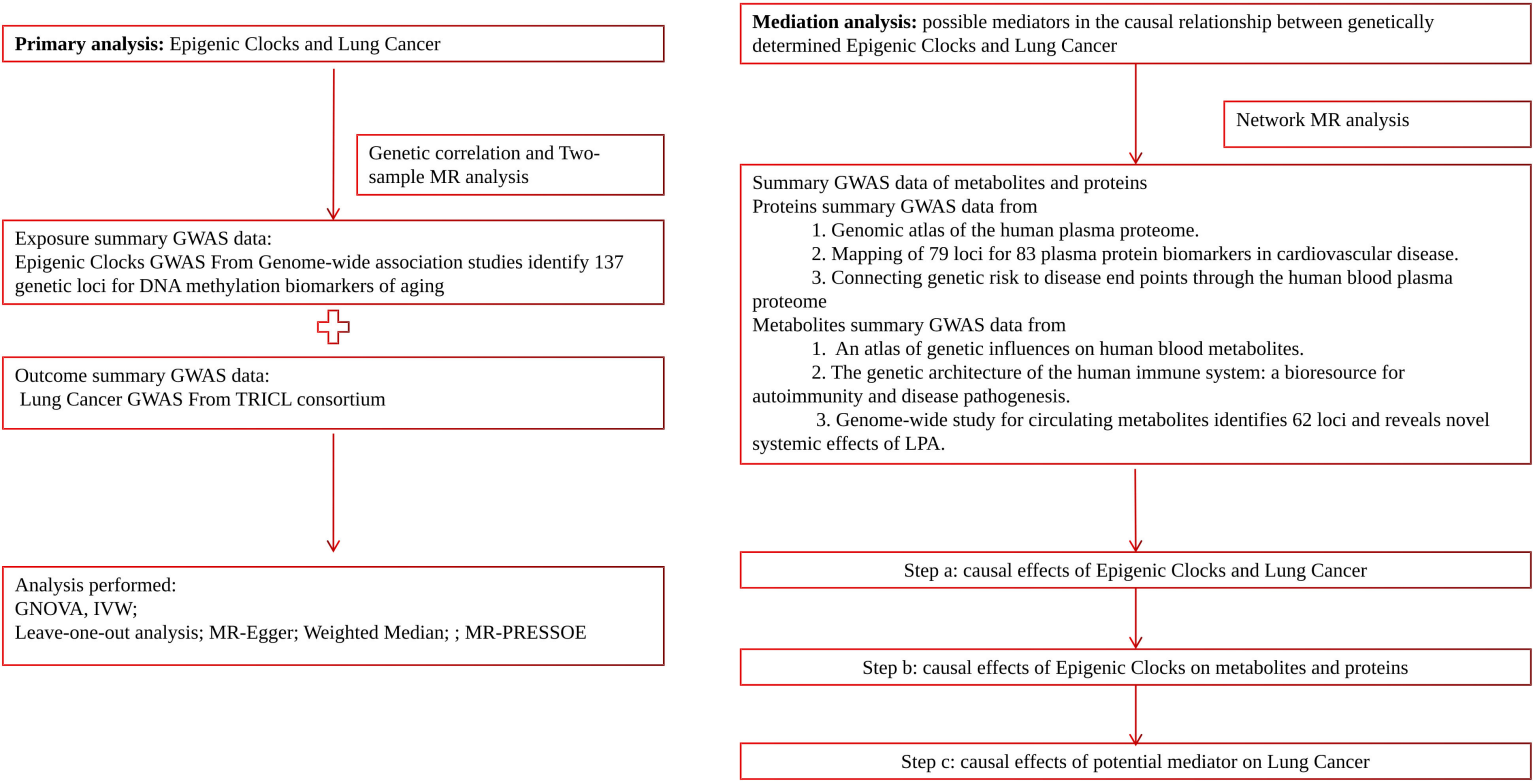
Schematic diagram of this study.

In this study, we employed a range of complementary genetic approaches, covering genetic correlation and Mendelian randomization, to comprehensively explore the causal relationship between epigenetic clocks and lung cancer, using large-scale GWAS summary statistics. Additionally, incorporating transcriptome and proteomics data into this study using network MR design may provide valuable insights into disease mechanisms and potential therapeutic targets. A flow chart detailing our study design can be found in **Figure 1**.

## 2 Methods

This study utilized a genome-wide association study (GWAS) data of four epigenetic clocks, including HannumAge, PhenoAge, and GrimAge, as well as epigenetic surrogate markers to assess their correlation with cancer risk (12). And single nucleotide polymorphisms (SNPs) were extracted from large-scale GWAS databases in the TRICL consortium (ieu-a-987, ieu-a-986, ieu-a-985). In this GWAS, TRICL consortium classified lung cancer patients as follows smoking, ever smoking, and never smoking. Finally, 85449 (smokers), 9298 (never smokers), 40453 (ever smokers) were identified for subsequent analysis. For blood proteins data (**Table S1**), we mainly used protein GWAS data published by Sun BB (1352 proteins), Folkersen L (2352 proteins), and Suhre K (352 proteins) (13–15). For blood metabolites data (**Table S2**), we mainly used protein GWAS data published by Shin SY (1352 metabolites), Roederer M (2352 metabolites), and Kettunen J (352 metabolites) (16–18).

### 2.1 Genetic correlation analysis

To assess the contribution of SNPs to the heritability of Epigenetic clocks and lung cancer, we employed GNOVA. This involved regressing the product of z-statistics obtained from two independent studies of these traits, which were derived from LD scores precomputed using 1000 Genomes European data (19, 20). Through this approach, we estimated both the SNP heritability (h2) of Epigenetic clocks and lung cancer and the overall genetic correlation (rg) between them.

### 2.2 Mendelian randomization design

Firstly, to ensure a strong correlation between IVs and Epigenetic clocks, we selected IVs significantly relationship with Vitamin (*P* < 5×10^−8^, *r* ^2^ < 0.001, genetic distance = 10000KB, minor allele frequency > 0.01) at the genome-wide level. Secondly, to satisfy the independence of genetic variation and confounders, we searched in Catalog and PhenoScanner databases to ensure that each IVs included was unrelated to known confounders. Finally, we calculated the F statistic to avoid the bias of weak IVs and ensured that these results were not affected by weak IVs (21, 22).

### 2.3 Mendelian randomization analyzes

Multiple Mendelian randomization (MR) methods were employed in this study, including inverse-variance weighted (IVW), maximum likelihood, MR using robust adjusted profile score (MR-RAPS), MR multivariate residual and outlier test (MR-PRESSO), MR-Egger, and weighted median, to estimate the causal effects of epigenetic clocks on cancer (21–25).

IVW was firstly employed to evaluate the potential impact of epigenetic clocks on tumorigenesis (22). The fixed-effect model was used in the absence of heterogeneity, while the random-effect model was adopted in case of heterogeneity. Subsequently, maximum likelihood estimation, MR-RAPS, MR-PRESSO, MR-Egger, and weighted median were utilized to further elucidate the relationship between epigenetic clocks and cancer. The objective of maximum likelihood estimation was to describe the distribution of probabilities by maximizing the likelihood ratio with low standard error (23). MR-PRESSO was essentially a variation of IVW, which could eliminate indicators that differed from causal associations with other IVs. Accurate results could be provided by MR-PRESSO when the horizontal pleiotropy existed in less than half of the IVs. On the other hand, MR-RAPS was relatively robust for various pleiotropic effects (25).

### 2.4 Heterogeneity and pleiotropy analysis

To ensure the third MR hypothesis that the IVs were independent of the outcome except for exposure, we used different methods for assessing potential effects. Cochran Q statistics and MR-Egger regression was used to explain the heterogeneity and pleiotropy in this study (26, 27). Then, the MR-PRESSO, leave-one-out analysis, and funnel plot were also used as additional multiplicity controls to global, outlier, and distortion tests.

### 2.5 Mediation analysis

This study was to explore potential mediating pathways of specific blood metabolites and proteins in the causal pathway from the epigenetic clock to cancer. We used a Mendelian randomization (MR) design with three independent steps (a-c) (28):

Step a: We first estimated the causal effect of the epigenetic clock, determined by genetics, on cancer. This step was similar to our preliminary analysis.

Step b: We utilized independent single nucleotide polymorphisms (SNPs) encoding protein-coding genes to estimate the causal effects of blood metabolites and proteins in the described GWAS summary data.

Step c: For any potential mediators with a causal relationship identified in Step b, we employed the inverse variance weighted (IVW) method to undertake one-to-one assessments.

If we found a clear causal relationship in all three steps, we could infer that specific blood metabolites and proteins mediated the pathway linking the epigenetic clock with cancer. Next, we derived the indirect effect of the epigenetic clock on cancer through each mediator by multiplying the results of Steps b and c. Finally, we estimated the weight of each mediator by dividing the mediated effect by the total effect.

### 2.6 Ethical approval

The sampling procedures, diagnostic criteria, quality control measures, and imputation techniques were delineated in their respective publications, and each GWAS study design does not require further ethical approval.

## 3 Results

### 3.1 Genetic correlation analysis

**Figure 2:**
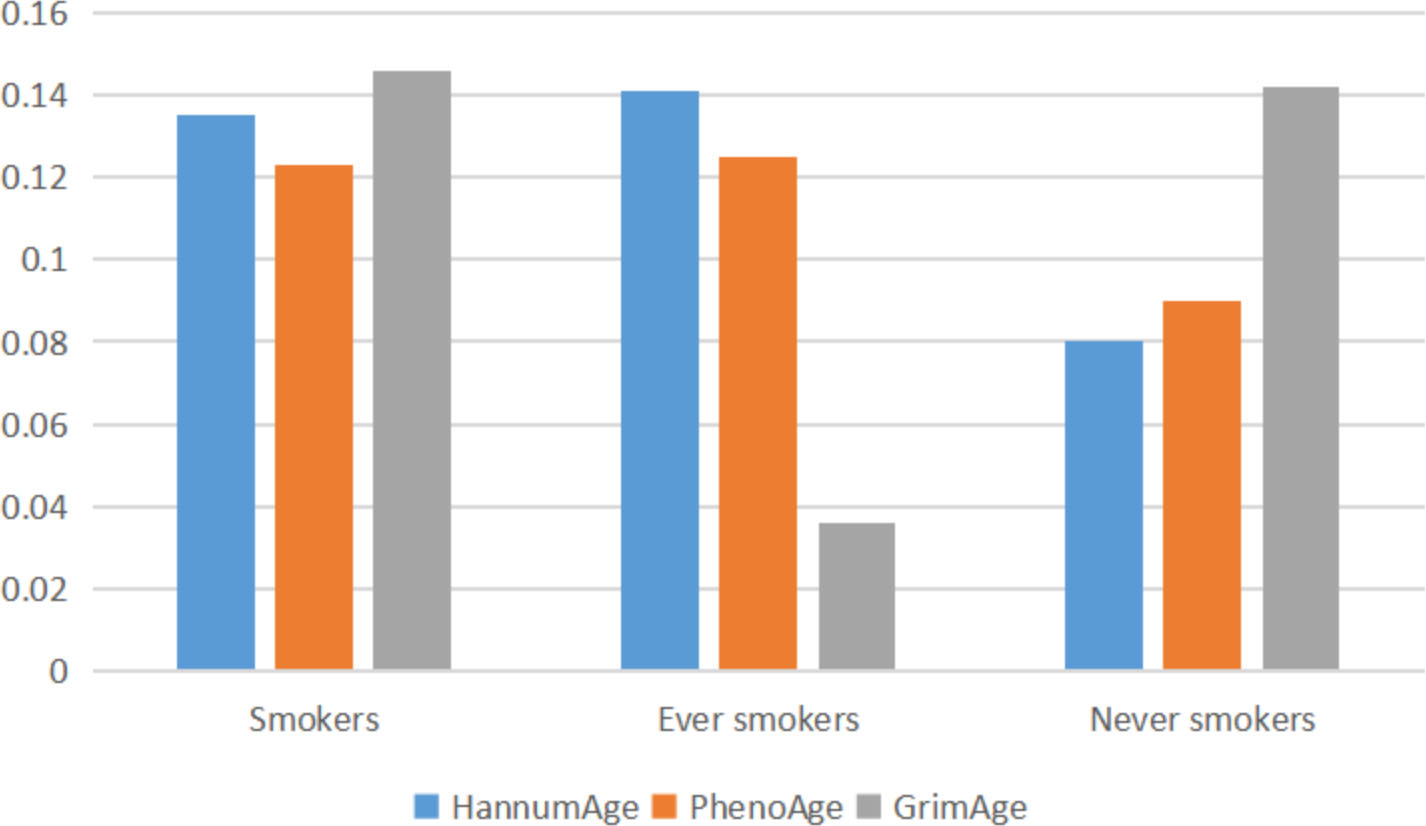
Correlation between epigenic clocks and lung cancer.

Through GNOVA analysis, we found there was significant genetic correlation implying shared genetic architecture between HannumAge and smokers (rg=0.135, *P*=1.080×10^-5^), ever smokers (rg=0.141, *P*=4.745×10^-5^) in lung cancer (**Figure 2**). However, we did not find a common genetic architecture between Horvath Intrinsic Age, PhenoAge, and GrimAge and lung cancer. Therefore, in the following analysis, we mainly analyzed the relationship between HannumAge and smokers, ever smokers (**Figure 2**).

### 3.2 Genetic instrumental variables for HannumAge

In this MR, 11 LD-independent IVs (after the clumping process) associated with HannumAge were included. The F statistics of each IV included were more significantly than 19.751, suggesting that we effectively excluded the effect of weak Ivs, which made these results more stable (**Table 1**).

**Table 1:** Genetic instrumental variables for HannumAge.

CHR	BP	BETA	SE	*P*	F
1	169549040	0.256626	0.0526221	5.38E-17	23.78290813
3	160301772	0.173412	0.0382163	6.83E-10	20.59022153
4	103468518	0.231341	0.0355169	9.21E-13	42.42629546
5	1285974	0.254141	0.0408623	3.58E-09	38.68154864
6	31196862	0.26506	0.0390643	6.07E-09	46.03925573
7	130416394	-0.288833	0.047146	3.71E-13	37.53219107
9	6448912	0.230314	0.0396861	1.96E-09	33.67936009
10	38216363	-0.355469	0.0566447	1.91E-17	39.38083341
10	49675247	-0.290248	0.0364699	7.26E-11	63.33872644
10	98052109	-0.536137	0.0727629	1.91E-17	54.29146493
11	66076360	0.163407	0.0367681	5.79E-10	19.75144789

CHR, chromosome; BP, base pair; SE, standard error.

### 3.3 MR analysis of HannumAge and lung cancer

**Figure 3:**
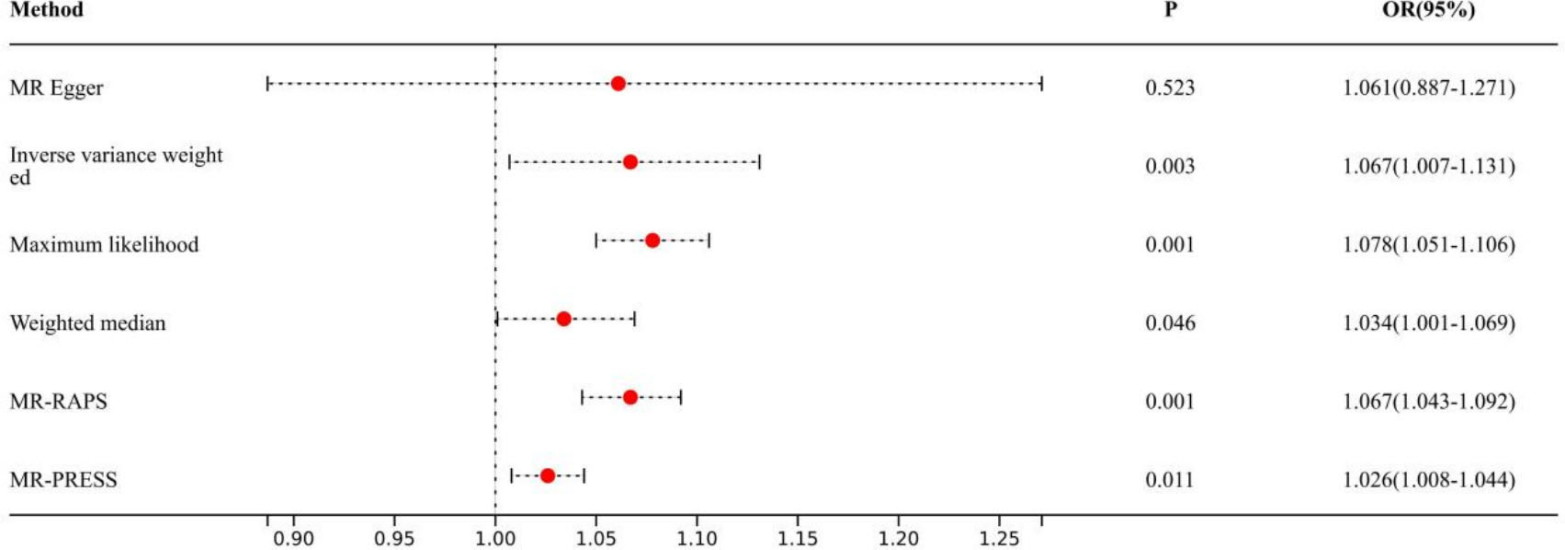
Association of epigenic clocks with lung cancer (smokers) in two-sample Mendelian randomization.

**Figure 4:**
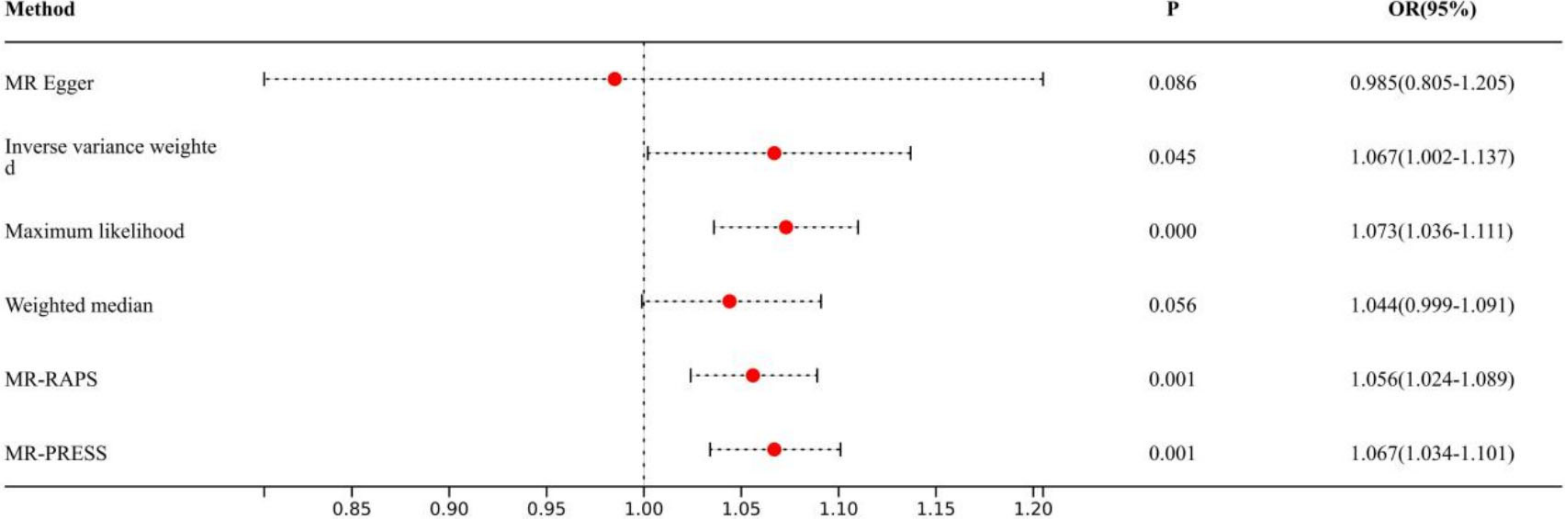
Association of epigenic clocks with lung cancer (ever smokers) in two-sample Mendelian randomization.

Causal effects are defined as odds ratio (OR) and can be interpreted as the logarithmic increase in the odds of lung cancer among current or former smokers with increasing HannumAge. For instance, using the IVW method, the OR estimate for current or former smokers with HannumAge and lung cancer was 1.067 (OR: 1.067, 95%CI: 1.007-1.131), suggesting that the average risk of developing HannumAge-related lung cancer is increased by 6.7% (**Figure 3**). We further found compelling evidence of a causal relationship between HannumAge and lung cancer (OR: 1.067, 95%CI: 1.002-1.137) (**Figure 4**). Additionally, estimates of the causal effect of HannumAge on lung cancer among current or former smokers using other MR methods were nearly the same as those obtained from the IVW method. Finally, while results from MR-Egger did not show a significant causal relationship between HannumAge and lung cancer, they suggest a positive correlation between the two.

To further evaluate the presence of heterogeneity and pluripotency in our MR analysis, we employed a series of methods. The Cochran’s Q test revealed significant heterogeneity in smokers (*P*=0.001), thus we used a random-effects model to account for heterogeneity and conducted further analysis. Remarkably, the consistent results were obtained (OR: 1.067, 95%Cl: 1.007-1.131 vs. OR: 1.026, 95%Cl: 1.008-1.044). Similarly, we found significant heterogeneity in ever smokers (*P*=0.001), so we again used a random-effects model to account for heterogeneity and found consistent results (OR: 1.067, 95%Cl: 1.002-1.137 vs. OR: 1.056, 95%Cl: 1.024-1.089). We also employed the MR-Egger regression, which supported that MR analysis was not impacted by horizontal pleiotropy (*P*=0.950, 0.426). Moreover, MR-PRESSO analysis revealed that the included instrumental variables (IVs) did not have significant outliers. Finally, the leave-one-out sensitivity analysis and funnel plot indicated that each included IV did not significantly impact our study results.

### 3.4 The potential mediator role of blood metabolites and proteins

**Figure 5:**
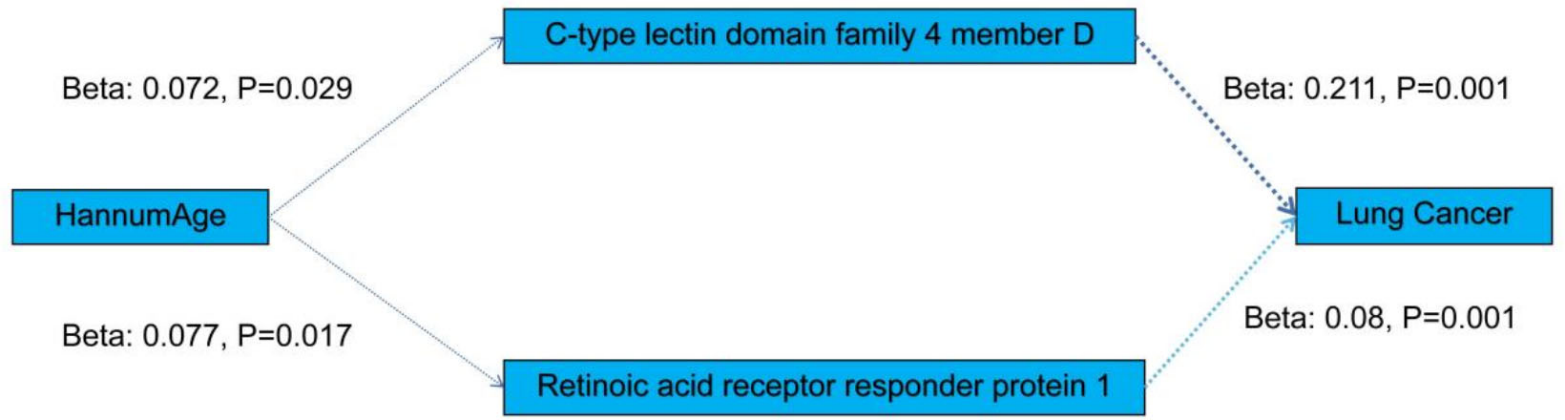
The potential pathogenesis in this study.

**Table S3** displays the causal relationship between HannumAge - a gene-based predictor of age - and blood metabolites and proteins, assessed using the MR method. A significant positive correlation was observed between the two. Further evaluation of whether blood metabolites and proteins have a causal relationship with cancer is presented in **Tables S4**–**S6**. We found Retinoic acid receptor responder protein 1, C-type lectin domain family 4 member D was significantly positively associated with lung cancer. Finally, we consider blood metabolites and proteins as risk factors as sleep apnea. And we speculate on the potential pathogenesis as shown in **Figure 5**.

## 4 Discussion

Our study represents the first application of complementary genetic approaches and mediator MR to investigate the potential relationship between cancer and the epigenetic clock. The results demonstrate a strong association between HannumAge and increased risk of cancer. Furthermore, mediator MR analysis reveals that retinoic acid receptor responder protein 1 and C-type lectin domain family 4 member D play a critical mediating role in the causal pathway from HannumAge to cancer, which suggesting that retinoic acid receptor response protein 1 and C-type lectin domain family 4 member D may serve as a potential drug target for the treatment of lung cancer

In order to establish causal relationships in our study, it was essential to anticipate and address any methodological deviations, and to ensure consistency with previous research. Therefore, we first compared our results with those of existing observational studies and conducted a reliability assessment. Our study results on the relationship between HannumAge and cancer were consistent with Li et al., 2022 prospective cohort study, which revealed a strong dose-response relationship between epigenetic clock and cancer risk (29). Similarly, a case-control study of lung cancer (n=332) reported a strong positive correlation (Beta=0.13, P<2.2×10^-16^) between methylation and both age and lung cancer risk (30). In addition, a 2019 study demonstrated a significant correlation between standardized measurements of rapid epigenetic aging and cancer incidence (HR: 1.50, P=3.4×10^−3^) (10).

The exact mechanism by which epigenetic clocks mediate lung cancer remains unclear. However, we speculate that they may influence cancer risk through two mechanisms. Firstly, evidence suggests that the association between cancer and age may be due to the accumulation of difficult-to-repair damage caused by exposure to carcinogens, such as those found in cigarette smoke (31). Levine et al. found a significant correlation between epigenetic clocks and cancer incidence in current smokers (*P*=7.4×10^−3^) and former smokers (*P*=0.039) (10). In our study, we observed a positive correlation between epigenetic clocks and lung cancer risk in patients who smoked or had previously smoked, which suggests that smoking may be an even larger risk factor for individuals with accelerated aging phenotypes. Although the mechanism underlying the co-occurrence of lung cancer and epigenetic clocks with cigarette smoke remains elusive, numerous genome-wide association researches have indicated associations between the 5p15 and 15q25 regions and cancer risk among smokers (32–34). Given the strong heritability of epigenetic age acceleration, these findings suggest the possible existence of inherent differences in susceptibility to endogenous stressors (35). Thus, further exploration of the association between gene loci implicated in lung cancer risk and lifespan in smokers and epigenetic clocks may have important clinical implications. If confirmed, epigenetic clocks could serve as useful markers for targeting cessation interventions.Secondly, some studies have suggested that abnormal immune system function and accelerated cellular aging may also be linked to lung cancer risk (36). With increasing age, the immune system may be more susceptible to dysfunction, leading to decreased immune surveillance and increased cancer risk (36). However, the exact pathophysiological mechanisms driving these associations remain to be elucidated.

In study, the results of mediator MR imaging showed that Epigenic Clockscould increase the concentration of proteins such as C-type lectin domain family 4 member D (CLEC4D), Retinoic acid receptor responder protein 1, leading to lung cancer.

CLEC4D, also known as DCIR (Dendritic Cell Immunoreceptor), is a protein belonging to the C-type lectin receptor family. It is primarily expressed on the surface of dendritic cells and macrophages, which are important components of the immune system. This receptor is involved in regulating immune responses, particularly in the context of antigen presentation and activation of immune cells (37). In the field of cancer research, CLEC4D has garnered attention due to its potential role in tumor immunity and cancer progression. A number of studies have investigated the relationship between CLEC4D and cancer. For example, in hepatocellular carcinoma (HCC), increased expression of CLEC4D has been observed and correlated with poor prognosis and tumor invasiveness (38). One possible mechanism is that CLEC4D enhances the migration, invasion, and metastasis of tumor cells by promoting epithelial-to-mesenchymal transition and activating signaling pathways involved in tumor progression (38). The exact mechanisms by which CLEC4D contributes to cancer development are still not fully understood, but they may involve its interactions with various ligands and complex signaling networks (39). Additionally, the expression and function of CLEC4D may be influenced by genetic and epigenetic changes, as well as the tumor microenvironment, further suggesting its involvement in cancer through Epigenetic Clocks mediating CLEC4D expression (40). However, extensive research is still needed to fully understand the mechanisms of action of CLEC4D in cancer and its potential as a therapeutic target. By elucidating its exact function and signaling pathways, strategies can be developed to modulate CLEC4D activity for therapeutic purposes.

Notably, compared with previous studies, this study has many strengths. First, with the help of complementary genetic and mediator MR methods, this study assessed the causal relationship of Epigenetic Clocks with lung cancer. Furthermore, we synchronized the use of multiple models based on different hypotheses to prevent erroneous reporting resulting from faulty assumptions in a single model.

Several limitations should be acknowledged in our study. Firstly, the study was restricted to a European population, which may restrict generalization to other populations. Secondly, unobserved pleiotropies were not accounted for, as is common in other MR studies. Lastly, the IVW effect estimates may be prone to bias caused by the presence of horizontal pleiotropy within some IVs.

## 5 Conclusions

In conclusion, a possible causality of Senescence with lung cancer risk was observed in the present study. However, the exact mechanism of senescence with lung cancer is still unclear and more studies will be conducive to explore it.

## Data availability statement

The original contributions presented in the study are included in the article/**Supplementary Material**. Further inquiries can be directed to the corresponding author.

## Author contributions

XF: Conceptualization, Investigation, Writing – review & editing. JZ: Conceptualization, Investigation, Writing – original draft. DL: Data curation, Investigation, Writing – review & editing. XL: Data curation, Methodology, Writing – original draft. TH: Data curation, Methodology, Writing – original draft. BL: Writing – original draft.
