# Supplementary material for: Using proteomics and metabolomics to identify therapeutic targets for senescence mediated cancer: genetic complementarity method
Source: Front Endocrinol (Lausanne). 2023 Sep 8;14:1255889. doi: 10.3389/fendo.2023.1255889 (PMC10514473; doi:10.3389/fendo.2023.1255889)
Supplement: Supplementary file 1 [file DataSheet_1.docx]

Supplementary Material

# Supplementary Tables

The Supplementary Material for this article can be found **Supplementary Tables**.**Table S1**: Blood proteins data used in this study.Table S2: Blood metabolites data used in this study.**Table S3**: Mendelian randomization analysis in HannumAge.**Table S4**: Mendelian randomization analysis in Smokers.**Table S5**: Mendelian randomization analysis in Ever Smokers.**Table S6**: Mendelian randomization analysis in Never Smokers.
